# 1061. Clinical Phage Microbiology: Evaluating Phages for Biofilm-associated Prosthetic Valve Endocarditis

**DOI:** 10.1093/ofid/ofab466.1255

**Published:** 2021-12-04

**Authors:** Daniel Gelman, Shunit Coppenhagen-Glazer, Ronen Durst, Ran Nir-Paz, Ronen Hazan

**Affiliations:** 1 Hadassah-Hebrew University Medical Center, Jerusalem, Yerushalayim, Israel; 2 The Hebrew University of Jerusalem, Jerusalem, Yerushalayim, Israel; 3 Department of clinical Microbiology and Infectious Diseases, Hadassah-Hebrew university medical Center, Jerusalem 91120, Israel, Jerusalem, Yerushalayim, Israel; 4 Institute of Biomedical and Oral Research (IBOR), The Hebrew University, Jerusalem, Israel, Jerusalem, Yerushalayim, Israel

## Abstract

**Background:**

**Prosthetic valve endocarditis (PVE**) is a major treatment challenge associated with biofilm formation. It requires intensive infectious diseases consultations and prolonged therapy. Nevertheless, high mortality rates are reported even with timely diagnosis and optimal management. *Bacteriophage (phage) therapy*, the use of bacterial viruses as antimicrobial agents, has been suggested as a potential adjunctive treatment for PVE. This is due to the ability of lytic phages to synergize with antibiotics and to destroy biofilms. However, due to their high specificity, it is crucial to match the phages by *in-vitro* evaluations that simulate the clinical settings.

**Methods:**

In this study we demonstrate this matching using an *in-vitro* PVE model of vancomycin-resistant *Enterococcus faecalis* (VRE). We have looked at the ability of the phage EFLK1, alone or in combination with antibiotics, to destroy mature biofilms from a commonly used bioprosthetic valve. In addition, we tried to predict these effects using several *in-vitro* phage susceptibility assays.

**Results:**

We found that the phage EFLK1 presents a significant inhibitory effect against planktonic cultures of VRE, both alone or in combination with ampicillin or ceftriaxone. We then tested the effect of these combinations on mature biofilm grown on a standard 96-well plates. We found that the phage, or its combination with ceftriaxone, led to a two-log reduction in the bacterial viability. In contrast, the addition of ampicillin to the phage caused interference with this antibacterial effect. When tested against biofilm grown on a pericardial patch, the combination of EFLK1 and ceftriaxone was found most efficient. Finally, when tested on the whole bioprosthetic aortic valve, we found that the phage EFLK1 alone was even more efficient than its combination with ceftriaxone.

Biofilm Eradication from Bioprosthetic Aortic Valve

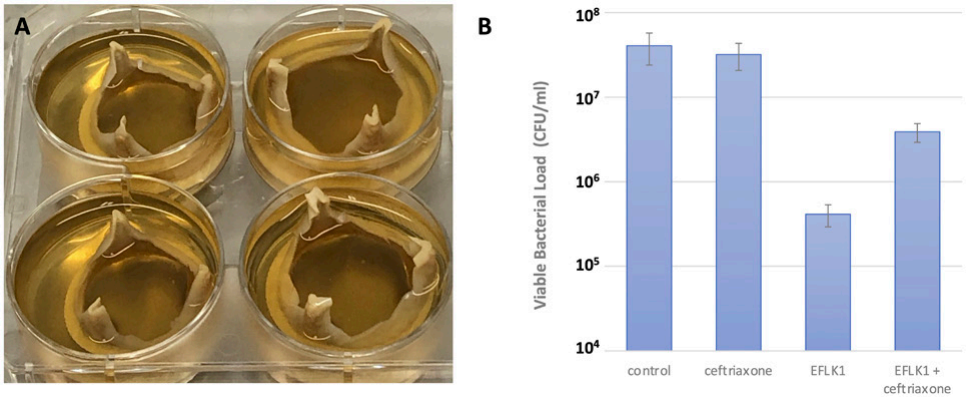

(A) Representation of E. faecalis biofilm formation on bioprosthetic valves. (B) Following 48-hours of growth, the valves were treated for five days by the phage EFLK1, ceftriaxone or their combination. The valves were then washed from any planktonic cells and the biofilm biomass was established by CFU enumeration.

**Conclusion:**

This study demonstrates that a proper *in-vitro* matching is essential in the treatment of PVE with phages. As seen here, the phage-antibiotic combination intended for treatment should be drawn according to their efficacy on suitable models, simulating the clinical settings, with the specific pathogen, the valve material, and the used phages taken into consideration.

**Disclosures:**

**Ran Nir-Paz, MD**, **BiomX** (Consultant)**Technophage** (Scientific Research Study Investigator, Advisor or Review Panel member)

